# Tsallis *q*-Statistics in Seismology

**DOI:** 10.3390/e25030408

**Published:** 2023-02-23

**Authors:** Leonardo Di G. Sigalotti, Alejandro Ramírez-Rojas, Carlos A. Vargas

**Affiliations:** Departamento de Ciencias Básicas, Universidad Autónoma Metropolitana-Azcapotzalco (UAM-A), Av. San Pablo 420, Colonia Nueva el Rosario, Alcaldía Azcapotazlco, Mexico City 02128, Mexico

**Keywords:** non-extensive statistical mechanics, Tsallis q-entropy, complex systems, seismicity, earthquakes

## Abstract

Non-extensive statistical mechanics (or *q*-statistics) is based on the so-called non-additive Tsallis entropy. Since its introduction by Tsallis, in 1988, as a generalization of the Boltzmann–Gibbs equilibrium statistical mechanics, it has steadily gained ground as a suitable theory for the description of the statistical properties of non-equilibrium complex systems. Therefore, it has been applied to numerous phenomena, including real seismicity. In particular, Tsallis entropy is expected to provide a guiding principle to reveal novel aspects of complex dynamical systems with catastrophes, such as seismic events. The exploration of the existing connections between Tsallis formalism and real seismicity has been the focus of extensive research activity in the last two decades. In particular, Tsallis *q*-statistics has provided a unified framework for the description of the collective properties of earthquakes and faults. Despite this progress, our present knowledge of the physical processes leading to the initiation of a rupture, and its subsequent growth through a fault system, remains quite limited. The aim of this paper was to provide an overview of the non-extensive interpretation of seismicity, along with the contributions of the Tsallis formalism to the statistical description of seismic events.

## 1. Introduction

The mechanism of earthquake generation involves a far from equilibrium process that cannot be described statistically in terms of classical extensivity. That is, it cannot be described in terms of classical thermodynamics by appealing to the properties of the Boltzmann–Gibbs entropy, which, being additive, is proportional to the number of elements of the system. In this case, the correlations within the system are essentially local. This is not the case with earthquakes, where the elements of the system are strongly correlated in time, space and magnitude [1,2,3,4,5,6,7]. In general, the established correlations are far from negligible at all scales, meaning that the probability of occurrence of a certain microstate depends on the occurrence of another microstate. In addition, earthquakes can be considered to be near critical, or even critical, phenomena exhibiting dynamic phase transitions [8], where a mainshock is the new phase. At, or near, the critical point, where phase transition (order–disorder) occurs, scaling laws with long-range order correlations are produced [9]. A similar situation occurs with earthquakes, where small seismic movements are more frequent than strong ones. Such systems, rather than obeying a Boltzmann distribution, are typically characterized by power-law behavior, which is then enhanced by long-range interactions or intermittency (i.e., large fluctuations) among the multiple accessible states [10]. Moreover, real seismicity is non-ergodic because, in general, the long-time average and the ensemble average of a physical quantity do not coincide.

In terms of the earthquake magnitude *M*, the cumulative distribution N(M), which provides the number of earthquakes with magnitude equal to, or greater than, *M*, has exponential behavior

(1) logN(M)=a−bM,

known as the Gutenberg and Richter (GR) law [11], where *a* quantifies the seismicity of a region and *b* is the slope of the cumulative distribution (which is close to 1 for tectonic earthquakes). The seismic energy dissipated (or seismic moment) by an earthquake can be related to the instrumental magnitude *M* as

(2) logE=cM+d,

where c=0.5 and d=9.05 [12]. These two expressions can be combined to produce the power-law relation for the number of earthquakes with seismic moments greater than *E* [13,14,15], namely

(3) $$N\left(E\right)∼E^{-\beta -1},$$

where the exponent β=2b/3 expresses the scale invariance contained in the dissipated seismic energy distribution [9,16]. Other parameters associated to seismic events, like the rate of aftershock production after a main event [17], the multifractal character of the temporal evolution of seismicity and the distribution of earthquake epicenters, also exhibit power laws [9]. In particular, the evidence of multifractality associated to earthquakes has motivated the conceptualization of the occurrence of earthquakes from a statistical mechanical approximation [10,16,18,19,20]. Although the GR law has been a classical standard measure for any model of earthquakes, the complexity of seismicity implies that earthquakes may be characterized by a much richer phenomenology. On the other hand, the frequency of earthquakes before and after the mainshocks is described by the Omori law [17,21]. In its modified form, the aftershock production rate between times *t* and t+dt after a mainshock at t=0 is given by

(4) $$dn\left(t\right)=K{\left(t+c\right)}^{-p},$$

where *K*, *c* and *p* are empirical constants with the exponent *p* taking values in the interval between 0.6 and 1.5 according to data from real seismic events [17], with a mean value slightly above unity in many cases. The proportionality constant *K* is a productivity that depends on the magnitude of the mainshock, while *c* is a case-dependent time scale, marking the onset of the power-law decay rate. This law, often called the Utsu–Omori law, states that the rate of aftershocks decreases hyperbolically with time. Both the GR and the Utsu–Omori law are power-law distributions with no characteristic scales, thereby stressing the complexity and criticality of real seismicity.

The time series that are recorded between successive earthquakes with arbitrary magnitudes exhibit a complex behavior that is characteristic of the statistics in time intervals of multifractal character [22,23,24], which is associated to the scale diversity of a fracture event in the Earth’s crust [25]. For example, seismic properties, such as the magnitude of an earthquake and the energy released during fracture processes, are known to behave dynamically in both the short- and long-term. As a consequence, these properties are robust to peculiar specific samples and are governed by the properties of scale symmetry, self-similarity and auto-affinity. However, it is not yet clear which of these properties would be of practical use for predicting the occurrence and magnitude of seismic events. Tsallis [26] introduced a generalization of the Boltzmann–Gibbs statistics with additive entropy into non-extensive statistical mechanics (NESM) through a non-additive entropy function $S_{q}$, which was later found to be successful in providing a statistical mechanical description of seismic events. NESM considers correlations at all scales between elements of the system and was inspired by multifractals and by imposing a convenient variational principle [27]. It has been attracting growing attention as a valuable tool to describe the statistical properties of a wide class of non-equilibrium complex systems [28,29], including seismicity [30]. In particular, in the last three decades since its inception, NESM has been quite successful in describing the macroscopic properties of earthquake events from the laboratory scale [31,32] to regional [33,34] and global scales [35]. Starting from the fragment–asperity interaction model, proposed by Sotolongo-Costa and Posadas, in 2004 [36], there has been a growing number of publications on seismicity, faulting, plate tectonics and precursory electromagnetic anomalies demonstrating the consistency between NESM and observations and providing further insight into the physics of earthquakes and the processes involved in the rupture initiation and growth through a fault system. In particular, good progress has been made in understanding the frequency–magnitude and energy distribution of seismicity, as well as the connection existing between the preparatory process of an earthquake and the temporal variation of the *q*-index [37,38,39,40,41]. On the other hand, the combination of non-extensivity with natural time and detrended fluctuation analysis has proved to be a powerful tool for the analysis of seismic data [42], while Tsallis entropy has also been employed in the analysis of precursory electromagnetic emissions [43]. Previous comprehensive reviews of non-extensive statistical seismology were reported by Vallianatos and co-workers [10,20,44], where fundamental properties and applications of NESM to seismology were also outlined.

In this study, we provide an overview of the non-extensive interpretation of real seismicity and the applications of concepts related to NESM in the description of the properties of faults and earthquakes. The paper is organized as follows. Section 2 introduces the non-additive entropy, $S_{q}$, and gives a brief account of the principles of NESM that emerge from it. The following sections deal with applications of NESM to seismology. In particular, Section 3 provides an overview of the non-extensive statistical aspects of earthquakes, starting with the fragment–asperity model, continuing with the frequency–magnitude and energy distribution of seismicity in different regions, the temporal variation of the *q*–entropic parameter, the spatio-temporal description of seismicity and closing with the non-extensivity of plate tectonics. Section 4 is devoted to the methodology resulting from combining NESM with natural time analysis for the examination of seismic data flutuations, while Section 5 deals with the implications of NESM on the precursory electromagnetic anomalies prior to an earthquake. Finally, Section 6 contains the relevant conclusions.

## 2. Non-Extensive Statistical Mechanics (NESM)

The heart of NESM lies in the generalization of the Boltzmann–Gibbs statistics to enable the study of systems with long-range interactions, long-term memories and/or multifractal structures. For such systems, Boltzmann–Gibbs statistical mechanics has a limited applicability, and its generalization, introduced by Tsallis [26], has been necessary to account for all length scale correlations among the elements of the system, leading to broad distributions with power-law asymptotic behavior. For a fully extended description of the theory the interested reader is referred to the papers in References [29,45,46] and the books by Abe and Okamoto [30] and Tsallis [28].

### 2.1. The $S_{q}$ Entropy

The non-additive $S_{q}$ entropy for a system composed of a discrete number of states is given by [28,47]

(5) $$S_{q}=\frac{k}{q-1}\left(1-\sum_{i=1}^{W}p_{i}^{q}\right),$$

for q∈R, where

(6) $$\sum_{i=1}^{W}p_{i}=1,$$

*k* is the Boltzmann constant of thermodynamics, $p_{i}$ is a discrete set of probabilities, *W* is the total number of microscopic configurations and *q* is a real number representing a measure of non-extensivity. The *q*-parameter is sometimes called the entropic index. When q=1, Equation (5) reduces to Boltzmann–Gibbs entropy

(7) $$S_{q=1}=S_{BG}=k\sum_{i=1}^{W}p_{i}\ln\frac{1}{p_{i}}.$$

For the case of equal probabilities, i.e., when $p_{i}=1/W$, ∀i, the Tsallis entropy takes the form

(8) $$S_{q}=\frac{k}{1-q}\left(W^{q-1}-1\right)=k\ln_{q}W,$$

where $\ln_{q}z$ is the *q*-logarithm, defined as

(9) $$\ln_{q}z\equiv \frac{z^{1-q}-1}{1-q},$$

which is, in turn, the inverse of the *q*-exponential function [28]

(10) $$\exp_{q}\left(z\right)\equiv {\left[1+\left(1-q\right)z\right]}^{1/(1-q)},$$

for 1+(1−q)z≥0 and 0 otherwise. Note that $\ln_{q=1}z\to \ln z$ and $\exp_{q=1}\left(z\right)\to \exp z$.

The non-additivity of $S_{q}$ is a manifestation of the fact that for two probabilistically independent systems, namely *A* and *B*, for which $p_{ij}^{A+B}=p_{i}^{A}p_{i}^{B}$, ∀(i,j), the entropy of the composite system satisfies the property

(11) $$S_{q}\left(A+B\right)=S_{q}\left(A\right)+S_{q}\left(B\right)+\frac{\left(q-1\right)}{k}S_{q}\left(A\right)S_{q}\left(B\right),$$

such that, if q<1 then $S_{q}\left(A+B\right)>S_{q}\left(A\right)+S_{q}\left(B\right)$ (super-extensivity), while if q>1, then $S_{q}\left(A+B\right)<S_{q}\left(A\right)+S_{q}\left(B\right)$ (sub-extensivity). It is precisely from this property that the term *non-extensivity* was coined [47]. It is straightforward that when q=1, Equation (11) reduces to the additive Boltzmann–Gibbs entropy for the composite system. Moreover, if *A* and *B* are correlated, a *q*-value may exist for which

(12) $$S_{q}\left(A+B\right)=S_{q}\left(A\right)+S_{q}\left(B\right),$$

so that $S_{q}$ is extensive for q≠1. The change of Tsallis entropy when two non-extensive systems with different temperatures, in contact with each other, reach thermal equilibrium was investigated by Du [48], who found that the principle of entropy increase of the composite non-extensive system after contact was verified, providing a generalization of the classical inequality $\Delta S_{BG}\geq 0$ in the context of Tsallis theory.

For a continuous variable z∈R, Tsallis entropy is expressed according to the integral formulation

(13) $$S_{q}\left(z\right)=\frac{k}{q-1}\left(1-\int_{0}^{\infty }p^{q}\left(z\right)dz\right),$$

where p(z)∈[0,1] is the probability distribution of *z*. Starting from the normalization condition of the probability distribution and the *q*-expectation value of the escort probability, $P_{q}\left(z\right)$, given by [33]

(14) $$P_{q}\left(z\right)=p^{q}\left(z\right){\left(\int_{0}^{\infty }p^{q}\left(z\right)dz\right)}^{-1},$$

with

(15) $$\int_{0}^{\infty }P_{q}\left(z\right)dz=1,$$

and, using the method of Lagrange multipliers, the optimal physical probability can be obtained as [10,20]

(16) $$p\left(z\right)=\frac{1}{y_{q}}\exp_{q}\left(-\beta_{q}z\right)=\frac{{\left[1-\left(1-q\right)\beta_{q}z\right]}^{1/(1-q)}}{y_{q}},$$

where $y_{q}$ is the *q*-partition function defined by the relation

(17) $$y_{q}=\int_{0}^{\infty }\exp_{q}\left(-\beta_{q}z\right)dz.$$

and

(18) $$\beta_{q}=\alpha_{2}{\left[\left(1-q\right)\alpha_{2}z_{q}+\int_{0}^{\infty }p^{q}\left(z\right)dz\right]}^{-1},$$

where $\alpha_{2}$ is a Lagrangian multiplier and $z_{q}$ is the *q*-expectation value [10,20]. Note that when q=1, $P_{1}\left(z\right)=p\left(z\right)$ and the *q*-expectation value of $P_{q}\left(z\right)$ takes the form of the standard expectation value of p(z).

### 2.2. The Cumulative Distribution Function and the *q*-Gaussian Distribution

In the framework of Tsallis theory, Abe and Suzuki [22,33] proposed that the escort probability is a more useful quantity than the physical probability, p(z), when comparing with observed distributions [28,49]. Therefore, the so-called *cumulative distribution function*, P(>z), can be better obtained upon integration of the escort probability, $P_{q}\left(z\right)$, rather than p(z). That is,

(19) $$P\left(>z\right)=\int_{0}^{\infty }P_{q}\left(z\right)dz=\exp_{q}\left(-\frac{z}{z_{0}}\right),$$

where $z_{0}>0$ and is defined by [50]

(20) $$z_{0}=\left(1-q\right)z_{q}+\frac{1}{\beta_{q}}.$$

Equation (19) can also be written in the alternative form

(21) $$\ln_{q}\left[P(>z)\right]=\frac{{\left[P(>z)\right]}^{1-q}}{1-q}=-\frac{z}{z_{0}},$$

which is the *q*-logarithm of the cumulative distribution function. This expression describes the distribution of the variable *z*. In fact, the *q*-logarithm of the cumulative distribution function is linear in *z*, with a slope equal to $-1/z_{0}$ [44]. When $z>z_{\min}$, the cumulative distribution of *z* becomes unity as long as $z=z_{\min}$, implying that Equation (19) must be replaced with the form [44,51]

(22) $$P\left(>z\right)=\frac{\exp_{q}\left(-\frac{z}{z_{0}}\right)}{\exp_{q}\left(-\frac{z_{\min}}{z_{0}}\right)},$$

in order to recover more consistency with real observations. In cases where $z_{\min}\ll z_{0}$ the above expression does not significantly change the estimated results [44].

Guided by the question of which distribution, other than that defined by Equation (19), can be used to perform comparisons with observed distributions, Tsallis [28] developed and described other possible forms. However, it was shown that these other forms were, indeed, all equivalent and one could be transformed into the other by means of simple algebraic operations involving *q* and $z_{0}$ [52,53,54,55]. An example was provided by Picoli et al. [54], who introduced the physical probability in the second constraint (or *q*-expectation value of *z*), namely,

(23) $$z_{q}=\int_{0}^{\infty }zp^{q}\left(z\right)dz,$$

to obtain the cumulative probability

(24) $$P\left(>z\right)={\left[1-\left(1-Q\right)\frac{z}{Z_{0}}\right]}^{1/(1-Q)},$$

where Q=1/(2−q) and $Z_{0}=\left(2-q\right)/z_{0}$. By applying these transformations to express the above probability in terms of *q* and $z_{0}$, the cumulative probability function is derived [55]

(25) $$P\left(>z\right)={\left[1-\left(1-q\right)\frac{z}{z_{0}}\right]}^{\left(2-q)/(1-q\right)}.$$

On the other hand, optimization of $S_{q}$ under the normalization condition of p(z) (referred to as the first constraint) and the second constraint, given by Equation (23) with p(z) replaced by the escort probability in terms of the squared variable $z^{2}$, leads to the generalization of the standard Gaussian distribution, known as the *q*-Gaussian distribution [28]

(26) $$p\left(z\right)=\frac{1}{y_{q}}{\left[1-\left(1-q\right){\left(\frac{z}{z_{0}}\right)}^{2}\right]}^{1/(1-q)},$$

where the standard Gaussian distribution is recovered in the limit q→1. For values of q>1, Equation (26) displays power-law tails with slope equal to −2/(q−1). This aspect enhances the probability of extreme events.

### 2.3. Tsallis q-Triplet

For a discrete system, Equation (16) takes the form

(27) $$p_{i}=\exp_{q}\left(-\beta E_{i}\right){\left[\sum_{j=1}^{W}\exp_{q}\left(-\beta E_{i}\right)\right]}^{-1},$$

where $E_{i}$ denotes the energy of the *i*th microscopic state and β is like an inverse temperature. In order to complete the above theory, the value of the entropic index *q* must be determined for a particular system. However, there are many examples showing that this value is actually hidden in the microscopic (or mesoscopic) dynamics of the system. An example, in terms of a family of logistic maps, is given by Tsallis and Brigatti [56]. For instance, according to Tsallis [57], there must be a *q*-triplet, i.e., $\left\{q_{\mathrm{stat}},q_{\mathrm{sen}},q_{\mathrm{rel}}\right\}$, for any complex system to be described by NESM. In particular, $q_{\mathrm{stat}}$ is the entropic index appearing in the maximum Tsallis entropy distribution, and, in the Tsallis entropy itself, $q_{\mathrm{sen}}$ characterizes the sensitivity of a non-linear dynamical system to the initial conditions, while $q_{\mathrm{rel}}$ is the index that controls the rate of relaxation and decay of correlation [58]. For the simple systems that are described by BG statistics $q_{\mathrm{stat}}=q_{\mathrm{sen}}=q_{\mathrm{rel}}=1$. The BG entropy principle is related to two-point Gaussian correlations, while the non-extensive Tsallis theory, which is a non-equilibrium statistical theory, is related to many-point correlations (i.e., long-range correlations) that are estimated by the functional derivative of the *q*-extended partition function. The Tsallis *q*-extension of the central limit theorem (CLT) produces a series of characteristic indices corresponding to different physical processes, the most significant of which are those defined by the *q*-triplet.

The non-extensive statistical mechanics is based, mathematically, on the non-linear differential equation [47]

(28) $$\frac{dy}{dx}=y^{q},\;\left[y=y(x)\right],$$

with y(0)=1 and q∈R. The solution of this equation is the *q*-exponential function, $\exp_{q}\left(x\right)$, defined by Equation (10). This *q*-extension of the CLT leads to the definition of the *q*-triplet. In particular, the non-linear differential equation

(29) $$\frac{d(p_{i}y_{q_{\mathrm{stat}}})}{dE_{i}}=-\beta q_{\mathrm{stat}}{\left(p_{i}y_{q_{\mathrm{stat}}}\right)}^{q_{\mathrm{stat}}},$$

describes a long-range-correlated, meta-equilibrium, non-extensive process and its solution corresponds to the probability distribution

(30) $$p_{i}=\frac{1}{y_{q_{\mathrm{stat}}}}\exp_{q_{\mathrm{stat}}}\left(-\beta_{\mathrm{stat}}E_{i}\right),$$

where $\beta_{\mathrm{stat}}=1/\left(kT_{\mathrm{stat}}\right)$ and the partition function is given by

(31) $$y_{q_{\mathrm{stat}}}=\sum_{i=1}^{W}\exp_{q_{\mathrm{stat}}}\left(-\beta q_{\mathrm{stat}}E_{i}\right),$$

where *W* is the total number of microscopic configurations. Using the *q*-exponential function defined by Equation (10), the probability distribution (30) becomes

(32) $$p_{i}∼{\left[1-\left(1-q_{\mathrm{stat}}\right)\beta_{q_{\mathrm{stat}}}E_{i}\right]}^{1/(1-q_{\mathrm{stat}})},$$

for discrete energy states $E_{i}$. The counterpart of Equation (32) for continuous states *z* of {Z}, having values that correspond to the state points of the phase space, can be written as

(33) $$p\left(z\right)∼{\left[1-\left(1-q_{\mathrm{stat}}\right)\beta_{q_{\mathrm{stat}}}z^{2}\right]}^{1/(1-q_{\mathrm{stat}})}.$$

In particular, the solutions p(z), given by the above distribution function, describe the probabilistic nature of the dynamics on the attractor set of the phase space. In addition, the non-equilibrium dynamics can evolve in different attractor sets, depending on the control parameters, and $q_{\mathrm{stat}}$ changes as the attractor set of the dynamics changes.

The entropy production is related to the profile of the attractor set of the dynamics, which can be described by its multifractality and by its sensitivity to the initial conditions, which can be expressed as

(34) $$\frac{d\psi }{dt}=\lambda_{1}\psi +\left(\lambda_{q}-\lambda_{1}\right)\psi^{q},$$

where the trajectory deviation in the phase space, ψ, is given by

(35) $$\psi \equiv \lim_{\Delta z(0)\to 0}\frac{\Delta z(t)}{\Delta z(0)},$$

and Δz(t) is the distance between neighboring values of *z* [59]. Equation (34) has the solution

(36) $$\psi \left(t\right)={\left\{1-\frac{\lambda_{q_{\mathrm{sen}}}}{\lambda_{1}}+\frac{\lambda_{q_{\mathrm{sen}}}}{\lambda_{1}}\exp_{q_{\mathrm{sen}}}\left[\left(1-q_{\mathrm{sen}}\right)\lambda_{1}t\right]\right\}}^{1/(1-q_{\mathrm{sen}})},$$

where the index $q_{\mathrm{sen}}$ is now related to the multifractal profiles of the attractor set through the expression

(37) $$\frac{1}{q_{\mathrm{sen}}}=\frac{1}{\alpha_{\min}}-\frac{1}{\alpha_{\max}}.$$

In the above relation $\alpha_{\max}$ and $\alpha_{\min}$ define the zero points of the multifractal spectrum f(α), i.e., $f\left(\alpha_{\max}\right)=f\left(\alpha_{\min}\right)=0$ [59]. Since these points can be measured, the entropic index $q_{\mathrm{sen}}$ can readily be calculated using Equation (37).

The theory of thermodynamic fluctuation–dissipation is based on Einstein’s original diffusion theory (i.e., the theory of Brownian motion), where the process of diffusion is, by itself, a mechanism for extremization of the entropy. If ΔS represents the deviation of entropy from its equilibrium value $S_{0}$, then the probability of a proposed fluctuation is ∝exp(ΔS/k). At a macroscopic level, the relaxation of some dynamical observable, F(t), related to the system evolution in the phase space to an equilibrium stationary state, is described by the linear differential equation

(38) $$\frac{d\Omega }{dt}=-\frac{\Omega }{\tau },$$

where

(39) $$\Omega \left(t\right)\equiv \frac{F(t)-F(\infty )}{F(0)-F(\infty )},$$

describes the relaxation of the macroscopic observable F(t) towards its stationary state value. On the other hand, the non-extensive generalization of the above classical theory is related to the general correlated anomalous diffusion process [56], where the equilibrium relaxation process is transformed to the meta-equilibrium, non-extensive relaxation process by the non-linear differential equation

(40) $$\frac{d\Omega }{dt}=-\frac{\Omega_{q_{\mathrm{rel}}}}{T_{q_{\mathrm{rel}}}},$$

which admits the solution in terms of the *q*-exponential function

(41) $$\Omega \left(t\right)=\exp_{q_{\mathrm{rel}}}\left(-\frac{1}{T_{q_{\mathrm{rel}}}}\right).$$

Candidate observables for the estimation of $q_{\mathrm{rel}}$ can be defined by the autocorrelation function or mutual information. Since the autocorrelation function captures the linear characteristics of the time series, mutual information, which is perhaps a much better function for capturing the non-linearity of the underlying dynamics, is usually preferred.

## 3. Non-Extensive Tsallis Theory in Seismology

In the last fifteen years, there has been a large number of applications highlighting the role of Tsallis theory as the correct statistical framework for the description of seismic events. For example, Tsallis theory succeeded in describing the spatio-temporal behavior of earthquakes and faults in a wide range of scales [20]. In general, earthquakes are complex physical systems with long-range correlations in space and time, long-term memory and/or multifractal structures, as opposed to physical systems exhibiting short-range correlations and short memory. Consideration of all-length scale correlations among the elements of the system has led to broader distributions with power-law asymptotic behavior. In addition, since in statistical physics the critical point is associated to power-law scalings and strong correlations, it has long been recognized that earthquakes are critical phenomena undergoing a phase transition [9,18,60].

### 3.1. The Sotolongo-Costa and Posadas Model for Earthquake Magnitudes

**Figure 1:**
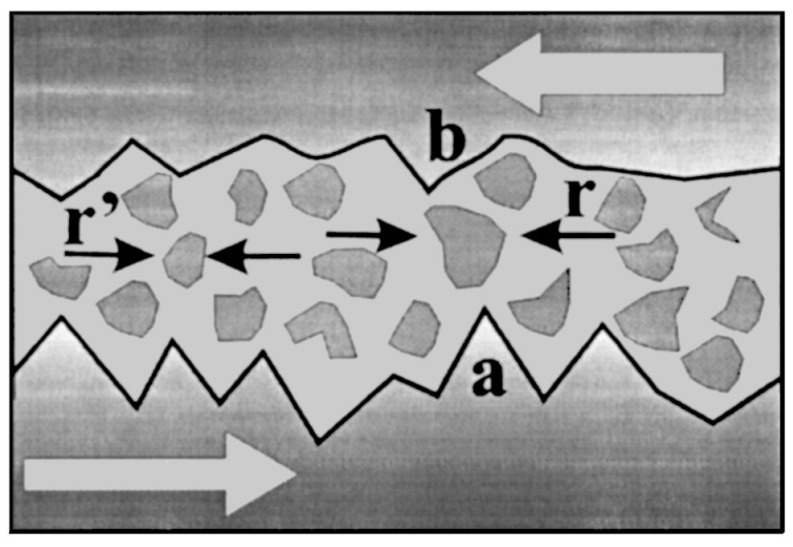
Schematic drawing showing two fault blocks with rough surfaces moving relative to fragmentary material between them. The motion of the blocks between points *a* and *b* in the figure is hindered by the presence of fragments. Here *r* and $r^{\prime }$ denote the size of the fragments. The white arrows indicate motion of the blocks and the black ones indicate motion of the fragments. From Sotolongo-Costa and Posadas [36] (their Figure 1).

The Sotolongo-Costa and Posadas model, also known as the fragment–asperity model for earthquake magnitudes, was introduced in 2004 by Sotolongo-Costa and Posadas [36]. This model describes the interaction between the irregularities of the fault blocks and the fragments between them as being the main causes of earthquakes (Figure 1). As the fragments hinder the relative displacement of the fault blocks, sufficient stress is accumulated until an asperity of the fault plane is broken or a fragment is displaced, resulting in energy release. In this sense, a realistic image of earthquakes is obtained by relating the fragment size distribution function, derived from a non-extensive statistical formulation, with the energy distribution of earthquakes. The probability p(σ) of finding a fragment of relative surface σ is related to the *q*-entropy, $S_{q}\left(\sigma \right)$, by means of Equation (13) with z→σ, subject to the normalization condition of p(σ) and the *q*-mean value, $\sigma_{q}$, given by Equation (23) with z→σ. Under the assumption that $\sigma_{q}=1$, the following expression was derived for the fragment size distribution function

(42) $$p\left(\sigma \right)d\sigma =\frac{{\left(2-q\right)}^{1/(2-q)}d\sigma }{{\left[1+\left(q-1\right){\left(2-q\right)}^{\left(q-1)/(2-q\right)}\sigma \right]}^{1/(q-1)}}.$$

As $\sigma ∼r^{2}$, and noting the released energy, ϵ∼r, so that $\sigma ∼\epsilon^{2}$, where *r* is the linear size of a fragment (Figure 1), Sotolongo-Costa and Posadas [36] deduced, from Equation (42), the expression

(43) $$p\left(\epsilon \right)d\epsilon =\frac{2C_{1}k\epsilon d\epsilon }{{\left[1+C_{2}k\epsilon^{2}\right]}^{1/(q-1)}},$$

for the energy distribution function, where $C_{1}={\left(2-q\right)}^{1/(2-q)}$, $C_{2}=\left(q-1\right){\left(q-2\right)}^{\left(q-1)/(2-q\right)}$ and *k* is the proportionality constant between σ and $\epsilon^{2}$. In this expression p(ϵ)=n(ϵ)/N, where n(ϵ) stands for the number of earthquakes of energy ϵ and *N* is the total number of earthquakes. The cumulative number N(>ϵ), i.e., the number of earthquakes with energy greater than ϵ is calculated according to

(44) $$N\left(>\epsilon \right)=N\int_{\epsilon }^{\infty }p\left(\epsilon \right)d\epsilon .$$

Since the earthquake magnitude, *m*, scales as ln(ϵ), Equations (43) and (44) can be combined to yield the expression for the cumulative number of earthquakes with magnitudes larger than *m*

(45) $$\ln\left[\frac{N(>m)}{N}\right]=\left(\frac{2-q}{1-q}\right)\ln\left[1+10^{2m}k\left(q-1\right){\left(2-q\right)}^{\left(1-q)/(q-2\right)}\right].$$

**Figure 2:**
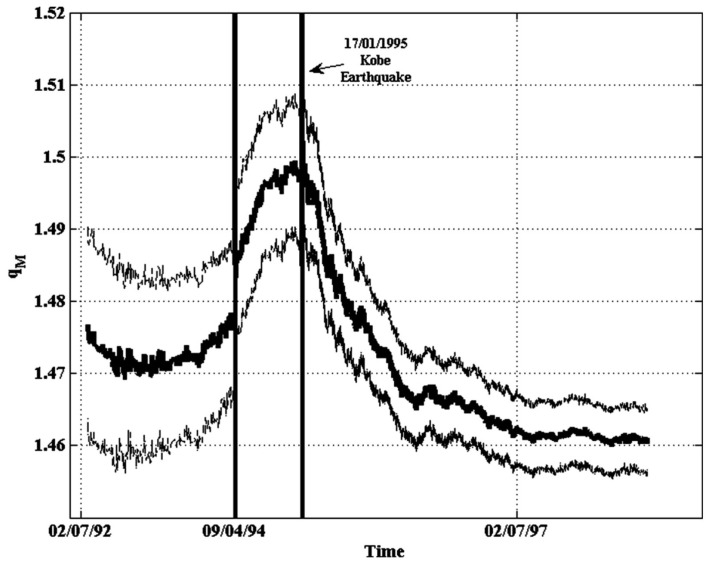
Temporal variation of the $q_{M}$-index (solid line) over increasing cumulative time windows and the associated standard deviation (dashed lines). The vertical line on the right marks the date of the Kobe earthquake (Japan). From Papadakis et al. [66] (their Figure 3).

This formula was found to fit very nicely the data from the catalogs of earthquakes in the Iberian Peninsula, and the Andalusian region and to the Californian earthquakes with magnitudes m>3 (see Figure 2 of Sotolongo-Costa and Posadas [36]). The *q* parameters from these catalogs were found to be q=1.65, 1.64 and 1.60 for seismicity in California, the Iberian Peninsula and the region of Andalusia, respectively, showing an intriguing similarity.

Later on, Silva et al. [61] proposed the scaling $\epsilon ∼r^{3}$ between the relative energy released and the volume of the fragments so that $\sigma -\sigma_{q}={\left(\epsilon /A\right)}^{2/3}$, where *A* is the proportionality constant between ϵ and $r^{3}$. This way, Equation (43) takes the form

(46) $$p\left(\epsilon \right)d\epsilon =\frac{C_{1}\epsilon^{-1/3}d\epsilon }{{\left[1+C_{2}\epsilon^{2/3}\right]}^{1/(q-1)}},$$

and the cumulative number of earthquakes becomes

(47) $$\frac{N(\epsilon >\epsilon_{\mathrm{thr}})}{N}=\int_{\epsilon_{\mathrm{thr}}}^{\infty }p\left(\epsilon \right)d\epsilon ={\left[1-\frac{\left(1-q\right)}{\left(2-q\right)}{\left(\frac{\epsilon_{\mathrm{thr}}}{A}\right)}^{2/3}\right]}^{\left(2-q)/(1-q\right)},$$

where now $N(\epsilon >\epsilon_{\mathrm{thr}})$ is the number of earthquakes with energy greater than the threshold value $\epsilon_{\mathrm{thr}}$. Using the earthquake magnitude m∼(2/3)ln(ϵ) in Equation (46), Telesca [37] derived the slightly different form of Equation (47)

(48) $$\frac{N(m>M_{\mathrm{thr}})}{N}={\left[1-\frac{\left(1-q\right)}{\left(2-q\right)}\left(\frac{10^{M_{\mathrm{thr}}}}{A^{2/3}}\right)\right]}^{\left(2-q)/(1-q\right)},$$

for the distribution of the number *N* of earthquakes with magnitude *m* larger than the threshold value $M_{\mathrm{thr}}$. A further function relating the cumulative number of earthquakes with the earthquake magnitude was derived by Telesca [7] to be

(49) $$\frac{N(m>M)}{N}={\left[\frac{1-\left(\frac{1-q_{M}}{2-q_{M}}\right)\left(\frac{10^{M}}{A^{2/3}}\right)}{1-\left(\frac{1-q_{M}}{2-q_{M}}\right)\left(\frac{10^{M_{\mathrm{thr}}}}{A^{2/3}}\right)}\right]}^{\left(2-q_{M}\right)/\left(1-q_{M}\right)},$$

where $q_{M}$ is the *q*-index related to the earthquake magnitude *M* and, as before, $M_{\mathrm{thr}}$ is the threshold magnitude. In passing, we note that many authors have pointed out that a rising value of $q_{M}$ is indicative of strong displacements of the fault blocks relative to the fragments, implying a transition away from equilibrium [62,63,64,65,66,67]. This suggests that the $q_{M}$-index can be used as a measure of the stability of an active seismic region. On the other hand, based on the entropy maximization principle, Darooneh and Mehri [38] derived a modified GR-law having the form of a *q*-stretched exponential. Under the assumption that the surface size of the fragments is distributed in the form of the escort probability, these authors derived the probability fragment size distribution

(50) $$p\left(\sigma \right)d\sigma =\beta \exp_{q}^{q}\left(-\beta \sigma \right)d\sigma ,$$

where

(51) $$\beta =\beta^{\prime }{\left[\left(1-q\right)\beta^{\prime }z_{q}+\int_{0}^{\infty }p^{q}\left(z\right)dz\right]}^{-1},$$

$\beta^{\prime }$ is a Lagrange multiplier and $z_{q}$ is the *q*-expectation value of *z* defined in terms of the escort probability

(52) $$z_{q}=\int_{0}^{\infty }zP\left(z\right)dz.$$

Inspired by the fractal nature of the fragments filling the gap between slipping fault blocks, Darooneh and Mehri [38] conjectured a relation of the form

(53) $$\epsilon ∼\exp\left(\sigma^{1/A}\right),$$

between the released relative energy and the size of the fragments. Using this new scaling in Equation (50) produces the probability seismic energy distribution

(54) $$p\left(\epsilon \right)d\epsilon =\beta \exp_{q}^{q}\left[-\beta {\left(\epsilon \right)}^{A}\right]\frac{A}{\epsilon }{\left(\ln\epsilon \right)}^{A-1}d\epsilon .$$

When the scaling m∼ln(ϵ) is used to replace the release energy ϵ by the magnitude *m*, the *q*-stretched cumulative distribution of earthquakes with magnitude greater than *m* is obtained as

(55) $$N\left(>m\right)=N\exp_{q}\left(-\beta m^{A}\right),$$

which also represents a non-extensive modification to the GR-law. Equation (55) was found to fit the data from the catalogs of the Iranian and Californian earthquakes more accurately than Equations (45) and (47) over the whole range of magnitudes [38]. Evidently, there is no doubt that the fragment–asperity model describes the earthquake frequency–magnitude distribution much better than the GR-law over all magnitudes [10,20,44].

### 3.2. Frequency–Magnitude and Energy Distribution of Seismicity

The fragment–asperity model has been applied to different earthquake catalogs to examine the frequency–magnitude distribution of seismicity [37,38,39,40,41,68,69]. In particular, similar $q_{M}$-values to those calculated by Sotolongo-Costa and Posadas [36] for different earthquake catalogs were also estimated for seismicity in different regions, including the Samambaia fault in Brazil ($q_{M}=1.60$ for 100 events), the New Madrid fault in the USA ($q_{M}=1.63$ for 173 events) and the Anatolian fault in Turkey ($q_{M}=1.71$ for 8980 events) [61]. Other estimations from the analysis of crustal seismicity in Taiwan indicated a value of $q_{M}=1.68$ [70], while the whole Italian seismicity could be characterized by non-extensive statistics with $q_{M}=1.66$ [62,63]. However, compared to this value, the estimated ones for the volcanic areas of Vesuvius and Etna were found to be smaller, corresponding to $q_{M}=1.47$ and 1.56, respectively [63]. An even lower value $q_{M}=1.39$ was reported by Vallianatos et al. [71] for the seismic unrest of the Santorini volcanic complex. Such smaller values are presumably due to the different intensity of seismic activity and the mechanism of earthquake generation in volcanic areas.

**Figure 3:**
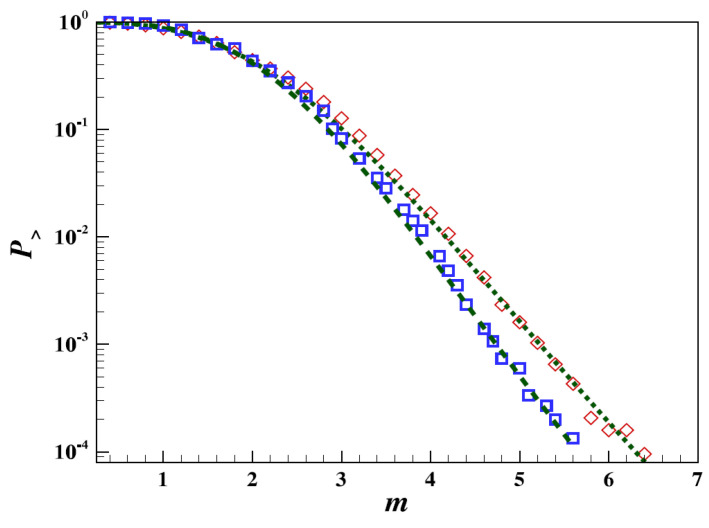
Cumulative distribution of the number of earthquakes as a function of magnitude for the seismicity in Iran (red squares) and California (blue diamonds). Fitting curves for the Iranian data (dashed line) and the Californian data (dotted line) were obtained using the q-stretched exponential cumulative distribution given by Equation (55). From Darooneh and Mehri [38] (their Figure 1). (Online version in color).

Using data from the strike–slip movement of the San Andreas fault, Vilar et al. [72] found that the best-fit *q*-index was given by $q_{M}=1.68$, which was, indeed, very close to the values estimated for other geological faults throughout the globe. For seismicity between 1960 and 2008 in the region of the Javakheti highlands in Georgia, Matcharashvili et al. [64] estimated a value of $q_{M}=1.80$. Moreover, Papadakis et al. [66] investigated the temporal variations of the $q_{M}$-index during the period 1990–1998 in regards to the 1995 earthquake in Kobe, Japan. The $q_{M}$ variations over increasing time windows using Equation (49), as shown in Figure 2, revealed that, on 9 April 1994, the $q_{M}$-parameter increased, reaching a peak value close to $q_{M}=1.50$ during the Kobe earthquake and decreasing afterwards to a value $q_{M}=1.46$, in 1997. An analysis of both the Iranian seismic data for the period between 1 January 1996, and 1 January 2005, and the Californian data for the period between 1 January 1932, and 1 January 2008, showed that the observed cumulative distribution of earthquake magnitudes fit better with the *q*-stretched exponential form (55) than with the forms given by the distributions (45) and (47), with standard errors that were factors of 1.7 (for the Iranian case) and 3.7 (for the Californian case) times lower [38]. Figure 3 shows the fitting of Equation (55) to the empirical data from the Iranian and Californian catalogs. In this figure, the dashed and dotted lines correspond to the best fitting of Equation (55) to the Iranian (red squares) and the Californian seismic data (blue diamonds), respectively.

Studies on the frequency–magnitude distribution of earthquakes in Greece revealed that the spatial distribution of $q_{M}$ was related to the spatial distribution of seismicity during the period 1976–2009 [67]. In particular, it was found that for shallow seismicity, i.e., for focal depth of ≤40 km, high $q_{M}$-values coincided with strong earthquakes. An analysis of the seismic activity in different zones of the Hellenic Subduction Zone also revealed that the $q_{M}$ variations were related to the seismic energy release in each zone [34]. A previous analysis of the $q_{M}$ variations prior to the 6 April 2009, L’Aquila earthquake (magnitude 5.8) showed a significant increase some days before the occurrence of the strong event [73]. Similarly, an increase of the $q_{M}$-index was also observed before the 12 October 2013, earthquake (magnitude 6.4) in the southwest segment of the Hellenic arc [74]. All these studies point to the fact that a significant increase of $q_{M}$ is associated to the occurrence of moderate-sized events prior to the mainshock, and, therefore, the increase must be taken as a preparatory phase leading to a strong earthquake, as is depicted in Figure 2 for the case of the 1995 Kobe earthquake. As shown by the seismogenic system of the North Aegean Trough (Greece), an increase of $q_{M}$ meant the existence of long-range correlations and only when its value significantly decreased did the system reach a state of equilibrium [75].

Telesca [37] applied a non-extensive analysis to the southern Californian earthquake data catalog by investigating only shallow earthquakes with depths of ≤60 km. For the minimum threshold magnitude $m_{0}=0$, he found that the best fitting of Equation (48) to the empirical data was obtained for $q_{M}=1.542$ and A=153.127, which were estimated using the maximum likelihood estimation method. In this case, deviations from the fitting curve from the normalized cumulative distribution function were found to be more significant at large magnitudes between 5.8 and 7.2. The best model was obtained for $m_{0}=2$, for which $q_{M}=1.506$ and A=438.65. Moreover, yearly values of *q* and *A* were calculated for the seismicity of southern California, during the period 1990–2010. Up to 2004, the *q*-values oscillated between 1.43<q<1.32 and increased afterwards, reaching a maximum value of 1.55 in 2010. In particular, the analysis revealed that the highest values of *A*, characterized by two significant spikes in 1992 and 1999 (see his Figure 6b), were indicative of events with the highest magnitude that occurred during those years. This is understandable, because the parameter *A* in Equation (48) represents the volumetric energy density and, therefore, its value is large only when the energy released is also large. On the other hand, the increasing trend of the *q*-index from 2004 to 2010, which coincided with a decreasing trend of *A* for the same period, was due to reduced seismic activity during those years, when only earthquakes with small magnitudes, between 4.5 and 5.5, occurred.

**Figure 4:**
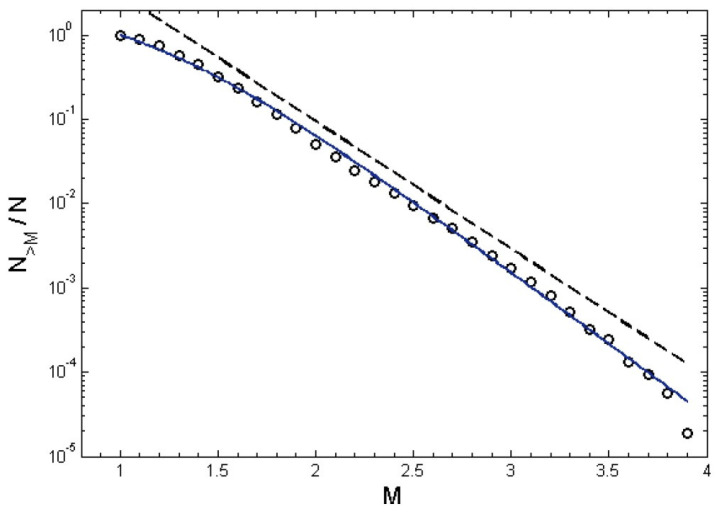
Normalized cumulative magnitude distribution function for $M\geq M_{\mathrm{thr}}=1$ (open circles) and the model of Equation (49) (blue solid line) for the seismicity of the West Corinth rift, Greece. The dashed line represents the GR scaling relation (1) for b=1.51. From Michas et al. [55] (their Figure 5a). (Online version in color).

Applications of the fragment–asperity model, using Equation (49), with the earthquake magnitude distribution data in the West Corinth rift were reported by Michas et al. [39,55]. In particular, Figure 4 shows the normalized cumulative magnitude distribution function for $M\geq M_{\mathrm{thr}}=1$ (open circles) and the fitted curve (blue solid line) according to Equation (49) for $q_{M}=1.37\pm 0.01$ and A=19.05±6.86 [55]. At lower earthquake energies, the model better described the observed distribution than the GR scaling relation (1) for b=1.51±0.03 (dashed line). Both the energy distribution function and the magnitude distribution were seen to decay as a power-law. The results of applying relation (49) as a function of magnitude, *M*, to seismic zones along the Hellenic Subduction Zone were reported by Papadakis et al. [34]. In this case, the cumulative number of earthquakes, as a function of magnitude, given by Equation (49), fit the observed data very well. A further application of this relation to the global seismic activity that occurred in Greece during the period 1976–2009 was reported by Antonopoulos et al. [68]. For the entire dataset, the model of Equation (49) described, very well, the observed data for M≥4.1 when $q_{M}=1.443\pm 0.018$ and $A=3.18\times 10^{5}\pm 1.7\times 10^{5}$, while, for the same dataset, but with the aftershocks removed, values of $q_{M}=1.46\pm 0.018$ and $A=3.25\times 10^{5}\pm 1.7\times 10^{5}$ were obtained for the best fit. This implied that removing or including the aftershocks in the data made little difference in the cumulative frequency–magnitude distribution for the seismicity in Greece for the period between 1976 and 2009.

**Figure 5:**
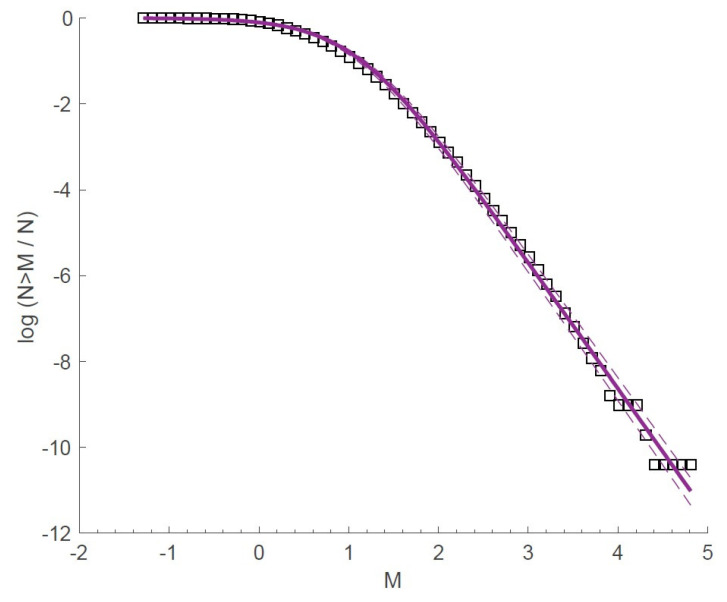
Frequency–magnitude distribution of the number of earthquakes as a function of magnitude for the seismicity in the volcanic field of the Yellowstone National Park for the period 1996–2016. The red solid line draws the best fit to the observed data (squares) using Equation (49). The dashed-line curves represent 95% confidence intervals. From Chochlaki et al. [41] (their Figure 10). (Online version in color).

More recently, Chochlaki et al. [41] investigated the magnitude–frequency distribution of earthquakes in the volcanic field of the Yellowstone National Park from 1996 to 2016. In particular, these authors studied the magnitude–frequency distribution of the released earthquake energies, *E*, resulting in the estimation of the $q_{E}$-index, expressing the long-term interactions of the system under the constraints presented by Sarlis et al. [42], which are reviewed later in Section 4. Application of Equation (49) to the 2008–2009 Yellowstone Lake earthquake swarm and the 2010 Madison Plateau earthquake swarm yielded $q_{E}=1.415\pm 0.01$ and A=257.9±41.5 for the former case and $q_{E}=1.496\pm 0.013$ and A=18.8±5.14 for the latter case. For both catalogs, Equation (49) fitted the observed data very well, suggesting that the fragment–asperity model provided the correct description of the seismic behavior and the energy release of the systems. Figure 5 shows the frequency–magnitude distribution of the number of earthquakes in the Yellowstone volcanic field during the period 1996–2016. The entropic parameter $q_{E}$ was estimated to be 1.44 from the fitting of Equation (49) (solid line) to the observed data (squares). This is an interesting case because, as Chochlaki et al. [41] argued, the Yellowstone seismicity was not only affected by its tectonic history, but also by the existing hydrothermal conditions, which could lead to high pore-pressure and pore-pressure diffusion associated with the network of fractures in the field. The spatial variation of $q_{E}$ through the volcanic field indicated values between 1.1 and 1.59, with the smallest values characterizing the seismicity in regions where several earthquake swarms had occurred in the past, such as in the northwest edge of the park in 1999, the Yellowstone Lake in 2008 and the Madison Plateau in 2010.

### 3.3. Temporal Variations of the Entropic Parameter

In the previous section, the time variation of the entropic parameters *q* and *A* was touched on only superficially for the case of seismicity in southern California [37]. In this section we delve further into the topic by reviewing the results from other authors in different regions. The variation of the *q*-index for the Greek seismicity was investigated by Michas et al. [39] for the period between 1980 and 2010 (see their Figure 3b). During this period the *q*-index was found to vary between 1.26 and 1.54 and to exhibit a clear correlation with the cumulative earthquake energy in each time interval (see their Figure 3c). This result demonstrated that the degree of non-extensivity was raised when both the *q*-parameter and the energy release increased, in which case the system moved away from equilibrium and larger earthquakes were expected. In this sense, the yearly variations of the *q*-index can be thought as encapsulating the seismic full history of a particular area or region. In the case of the Greek seismicity reported by Michas et al. [39], their Figure 3b,c indicate a periodicity of both the *q*-parameter and the released energy during the period 1980–2010. Within this time window increased activity was recorded from 5 to 7 years, followed by quieter periods lasting 7 to 10 years.

**Figure 6:**
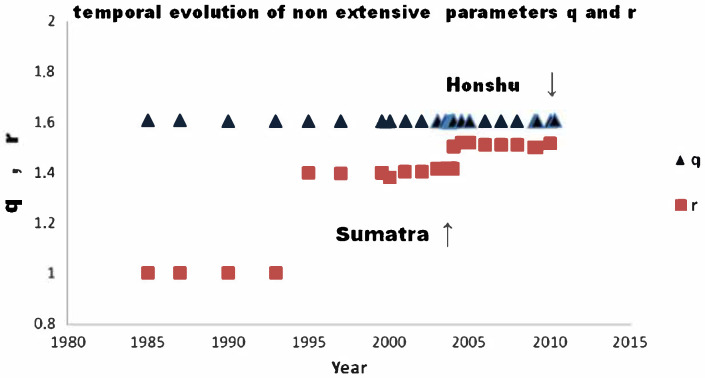
Time evolution of the non-extensive indices *q* and *r* regarding the preparatory process of the 2004 Sumatra–Andaman (magnitude 9.0) and the 2011 Honshu (magnitude 9.1) mega-earthquakes. From Vallianatos and Sammonds [35] (their Figure 2). (Online version in color).

The increase of $q_{M}$ is also related to an increase of the long-range spatial correlations, which drives the system toward a critical state leading to instability, large-scale reactions and energy release [43,73]. According to this view, strong earthquakes can be the result of large-scale correlations due to the redistribution of stresses over large-scales so that a rupture can arise as a consequence of a highly correlated stress field [73,76,77]. In order to interpret the physical processes that led to the 26 December 2004, Sumatra–Andaman (magnitude ≈ 9.0) and the 11 March 2011 Honshu Island, Japan (magnitude ≈ 9.1) mega-earthquakes, Vallianatos and Sammonds [35] employed the crossover formulation of non-extensive statistical mechanics [28,78] to examine the seismic moment distribution. In this formulation, the optimal physical probability (16) can be alternatively obtained by solving the non-linear differential equation

(56) $$\frac{dp}{dz}=-\beta_{q}p^{q},$$

which can be generalized to the form

(57) $$\frac{dp}{dz}=-\beta_{r}p^{r}-\left(\beta_{q}-\beta_{r}\right)p^{q},$$

where crossover to another type of behavior at larger values of *z* is observed [78]. This form reduces to Equation (56) when r=0 or r=q. For 1≤r<q, Equation (57) admits the solution

(58) $$\begin{array}{ccc} z=\frac{1}{\beta_{r}}\{\frac{p^{1-r}-1}{r-1} & - & \frac{(\beta_{q}/\beta_{r})-1}{1+q-2r}\left[H\left(1;q-2r,q-r,\left(\frac{\beta_{q}}{\beta_{r}}\right)-1\right)\right.\\ & - & \left.H\left(p;q-2r,q-r,\left(\frac{\beta_{q}}{\beta_{r}}\right)-1\right)\right]\}, \end{array}$$

where

(59) $$H\left(\xi ;a,b,c\right)=\xi^{1+a}F\left(\frac{1+a}{b},1;\frac{1+a+b}{c};-\xi^{b}c\right),$$

and *F* is the hypergeometric function. Here the *q*-index describes the seismic moment distribution of small and moderate earthquakes, while the *r*-index describes the seismic moment distribution of strong earthquakes. Figure 6 shows the time evolution of these two indices regarding the Sumatra and Hinshu mega-earthquakes. The *q*-index resulted in a constant value of 1.6, while the *r*-index varied from 1 to 1.5, meaning a jump from a BG exponential distribution (r=1) to a power-law distribution, where *r* was seen to vary from 1.4 to 1.5 during the preparatory process towards the mega-earthquake. This result supports the idea of a global organization of seismicity [35].

The complexity and self-organization that characterize the seismogenic system are associated to long-range interactions and long-term memory, which are collectively referred to as *correlation*.

**Figure 7:**
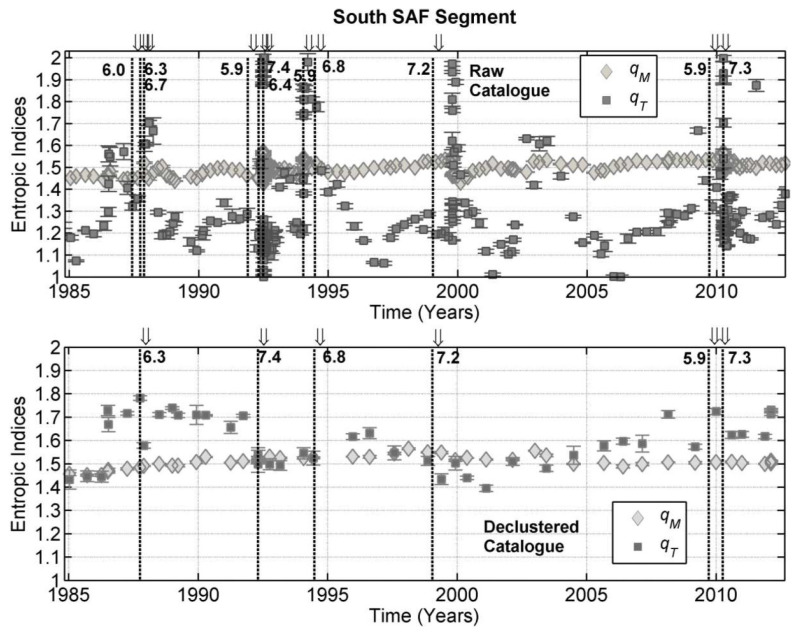
Time variation of the entropic indices $q_{M}$ and $q_{T}$ for the raw (**top**) and a declustered San Andreas Fault catalog (**bottom**). The error bars refer to 95% confidence intervals and the vertical dashed lines indicate the occurrence of earthquakes with local magnitude ≥5.9. From Efstathiou et al. [69] (their Figure 6).

Tzanis et al. [40] proposed that the problem could be addressed by constructing multivariate frequency distributions based on the formulation of joint distribution laws, where it is assumed that the distributions of the magnitude, *M*, and inter-event time, *T*, are statistically independent, in the sense that the joint probability P(M∪T) factorizes into the product P(M)P(T). Then, the empirical probability $P(>\left\{M\geq M_{\mathrm{thr}},T:M\geq M_{\mathrm{thr}}\right\})$ can be written as

(60) $$\begin{array}{ccc} \ln\frac{N(>\left\{M\geq M_{\mathrm{thr}},T:M\geq M_{\mathrm{thr}}\right\})}{N_{0}} & = & \ln{\left[1-\frac{\left(1-q_{M}\right)}{\left(2-q_{M}\right)}\left(\frac{10^{M}}{A^{2/3}}\right)\right]}^{\left(2-q_{M}\right)/\left(1-q_{M}\right)}\\ \; & + & \ln{\left[1-\left(1-q_{T}\right)\left(\frac{T}{T_{0}}\right)\right]}^{1/(1-q_{T})}, \end{array}$$

where $q_{T}$ is the entropic index for the inter-event times, $T_{0}$ is the *q*-relaxation time and $N_{0}$ is the total number of earthquakes at M=0. Equation (60) is a generalized (bivariate) GR law, in which $b_{q}=\left(2-q_{M}\right)/\left(q_{M}-1\right)$ [7] and corresponds to a natural description of earthquake thermodynamics. Tzanis et al. [40] implemented this model to the analysis of data from the north California earthquake catalog. They found raw catalog values of $q_{M}$ ranging from 1.43 at cut-off magnitudes $M_{c}=3.0$ to 1.53 at $M_{c}=4.4$, while the $q_{T}$-index varied between ≈1.3 for $M_{c}\leq 3.8$ and 1.4 at larger $M_{c}$, indicating a low to moderate correlation. For the declustered catalog $q_{M}$ was also found to exhibit a quasi-linear variation with a cut-off magnitude with $q_{M}=1.5$ for $M_{c}=3.0$ and 1.53 for $M_{c}=4.4$. However, the temporal entropic index $q_{T}$ was found to exhibit opposite behavior to its raw-catalog counterpart. That is, $q_{T}\approx 1.2$ for $M_{c}\leq 4$, indicating a low correlation, and then gradually dropping to about 1.1 at $M_{c}=4.4$, indicating only a weak correlation or, equivalently, a random temporal sequence of earthquake occurrence between magnitudes 4.2 and 4.3. A similar analysis was next conducted by Efstathiou et al. [69], who also employed the model of Equation (60) to examine the evolution of seismicity along the San Andreas Fault, California, starting at year 1980 and ending at year 2012. Figure 7 shows the time variation of the entropic indices for the raw (top) and stochastically declustered catalog (bottom). For instance, $q_{M}$ varied between 1.43 and 1.56 for the raw catalog with small localized fluctuations in time, corresponding to the occurrence of significant earthquakes. Similarly, for the declustered catalog $q_{M}$ took values between 1.45 and 1.56, with much smaller and slower variations. Conversely, for the raw catalog the $q_{T}$-index experienced stronger variations, being generally low, i.e., $q_{T}\approx$ 1.1–1.3 prior to large earthquakes and jumped to high values, between 1.8 and 2.0, after the incidence of large events, while for the declustered catalog $q_{T}$ varied less intensely between 1.40 and 1.78. For example, $q_{T}≳1.7$ up to the Landers event of mid 1992, then fluctuated to ≈1.5 before the Hector Mine earthquake of 1999 and, then, fluctuated again to values from ≈1.6 to 1.7 up to, and after, the Baja earthquake of 2010.

Figure 2 shows the time variation of the $q_{M}$-parameter during the period 1990–1998 for a broad area surrounding the epicenter of the 1995 Kobe earthquake [66]. The $q_{M}$ increased significantly prior to the strong earthquake on 9 April 1994, displaying the beginning of a preparatory phase several months before the Kobe earthquake. Quite interestingly, Papadakis and Vallianatos [75], through an analysis of the temporal variations of $q_{M}$ and *A* for the area of the North Aegean Trough during the period 1976–2015, found that after significant increase of $q_{M}$, coinciding with strong earthquakes of moment magnitudes >5, the value of $q_{M}$ did not significantly decrease after a mainshock, meaning that the seismic energy had not been fully released and, therefore, the degree of long-range correlations continued to increase and the system did not return to an equilibrium state.

### 3.4. Space–Time Description of Seismicity

The space–time properties of earthquakes have been studied for decades for the purposes of hazard assessment and forecasting. Such seismic attributes include the inter-event time and the inter-event distance. The inter-event time is defined as $T_{>M,k}=t_{k}-t_{k-1}$, where $t_{k}$ is the occurrence time of the *k*th event and $t_{k-1}$ is the occurrence time of the (k−1)th event in the catalog, with both events being of magnitude greater than *M* [79,80]. Another simple measure of separation between earthquake events is the so-called inter-event distance, which, according to Batac and Kantz [81], can be defined as

(61) $$D_{k}=R_{⊕}\cos^{-1}\left[\cos\left(\phi_{k+1}-\phi_{k}\right)\cos\left|\theta_{k+1}-\theta_{k}\right|\right],$$

where the spatial coordinates ϕ and θ correspond to the latitude and longitude coordinates (in radians), respectively, and $R_{⊕}=6371$ km is the approximate radius of the Earth. Note that this definition, which assumes a spherical surface and is based on epicenters, is just a special case of the general hypocentral separation distance used by Kagan and Knopoff [82].

The application of Tsallis entropy to the calculation of inter-event times and distances has been taken up by a handful of authors. For example, Abe and Suzuki [22,33] investigated the spatial and temporal properties of seismicity in California and Japan. In particular, they found that the distance between successive earthquakes obeyed a *q*-exponential distribution of the form given by Equation (19) and that $q_{D}<1$, where $q_{D}$ was the entropic index for the inter-event distances [33]. They also proposed the duality relation $q_{T}+q_{D}\approx 2$, where the cumulative inter-event time distribution, P(>T), for both regions, was also described by a *q*-exponential function with $q_{T}>1$ [22]. The dependence of the entropic indices $q_{M}$ and $q_{T}$ on the inter-event distance for the north California earthquake catalog was analyzed by Tzanis et al. [40]. They obtained values of $q_{M}$ between 1.46 and 1.52 over the range of inter-event distances 0<D<400 km. Conversely, the $q_{T}$-index underwent more significant variations with $q_{T}\approx 1.6$ for D<100 km and dropped to 1.3–1.4 at longer inter-event distances, implying the existence of long-range correlations in the mixed background-and-aftershock process. For the declustered catalog, $q_{M}$ was found to behave similarly to its raw counterpart and varied in the interval $1.48\leq q_{M}\leq 1.52$, while at D<150 km the temporal $q_{T}$-index varied between 1.41 and 1.52, implying a relatively high correlation due to the near-field interaction between nearby earthquake pairs. At greater inter-event distances $q_{T}$ was seen to drop to 1.2–1.37, implying a low to moderate correlation.

**Figure 8:**
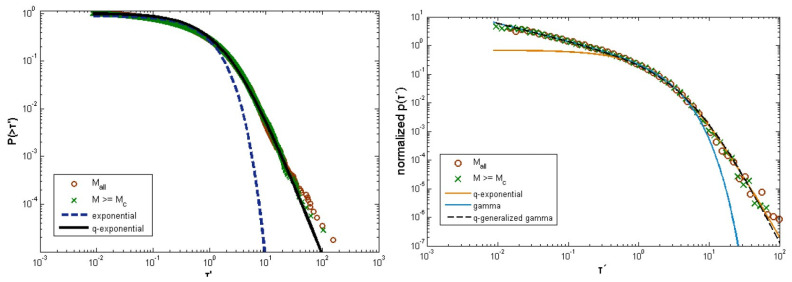
(**Left**) Cumulative distribution $P(>T^{\prime })$ as given by Equation (63) (solid line) and ordinary exponential (dashed line) for the entire dataset of the earthquake activity in the western Corinth rift (open circles) and for $M\geq M_{c}$ (crosses). (**Right**) q-exponential function, Gamma distribution as given by Equation (64) and q-generalized Gamma distribution as given by Equation (65) (dashed line) compared to the same observed data of the left frame. From Michas et al. [55] (their Figures 6a and 7b). (Online version in color).

The cumulative distribution functions of the inter-event times and distances were further estimated by Vallianatos et al. [83] for the 15 June 1995, Aigion earthquake aftershock sequence. They found $q_{T}=1.58$ and $q_{D}=0.53$ for the two distributions, respectively, so that $q_{T}+q_{D}=2.11$, was in good agreement with the findings of Abe and Suzuki [22,33]. On the other hand, Vallianatos and Sammonds [35] examined the inter-event time and distance distributions around the 2004 Sumatra–Andaman and 2011 Honshu mega-earthquakes, and found that $q_{T}=1.5$ and $q_{D}=0.3$, again supporting the conclusion of non-extensive spatio-temporal duality. Michas et al. [55] investigated the inter-event time distribution and the Gamma distribution for inter-event times for the earthquake activity at the West Corinth rift in central Greece. Starting from the cumulative distribution

(62) $$P\left(>T\right)=\exp_{q^{\prime }}\left(-B_{q^{\prime }}T\right),$$

associated to the physical probability $p\left(T\right)=\exp\left(-B_{q}T\right)/y_{q}$, with $q^{\prime }=1/\left(2-q\right)$ and $B_{q^{\prime }}=\left(2-q\right)/B_{q}$ [54], Michas et al. [55] employed the alternative form

(63) $$P\left(>T\right)={\left[1-\left(1-q\right)B_{q}T\right]}^{\left(2-q)/(1-q\right)},$$

which can be derived by applying the transformations for $q^{\prime }$ and $B_{q^{\prime }}$ to Equation (62). Normalization of the inter-event times *T* in seconds as $T^{\prime }=T/\tau$, where $\tau =\left(t_{N}-t_{1}\right)/\left(N-1\right)$ is the mean inter-event time, leads to the scaled $P(>T^{\prime })$. Moreover, these authors compared the performance of Equation (63) with the Gamma distribution function [84]

(64) $$p\left(T^{\prime }\right)=C{T^{\prime }}^{\left(\gamma -1\right)}\exp\left(-\frac{T^{\prime }}{\beta }\right),$$

which was shown by Corral [85] to be a universal one, since it holds for both local and regional scales and for a wide range of magnitudes, as long as the seismic activity is stationary, when C=0.5±0.1, γ=0.67±0.05 and β=1.58±0.15. However, the *q*-generalized Gamma function [86]

(65) $$p\left(T^{\prime }\right)=C{T^{\prime }}^{\left(\gamma -1\right)}\exp_{q}\left(-\frac{T^{\prime }}{\theta }\right),$$

was also employed in the comparison on the basis of the highly local character of the earthquake activity at the West Corinth rift, which leads to inter-event times that are highly non-random. The left frame of Figure 8 shows a log-log plot of $P(>T^{\prime })$ given by Equation (63) (for q=1.25±0.02 and $B_{q}=1.9\pm 0.3$; solid line) for the entire dataset (open circles) and for $M\geq M_{c}$ (crosses) as compared to the ordinary exponential distribution (dashed line), while the right frame depicts the *q*-exponential distribution p(T) for $y_{q}=1.47$, $B_{q}=1.34$ and q=1.23, the Gamma function given by Equation (64) for C=0.35, γ=0.39 and β=1.97, and the *q*-generalized Gamma distribution given by Equation (65) for C=0.35, γ=0.39, θ=1.55 and q=1.23. The cumulative distribution $P(>T^{\prime })$ given by Equation (63) fit the observed data very well, except for a small deviation for $T^{\prime }≳30$. For $M\geq M_{c}$ only less than 0.05% of the data actually deviates from the *q*-exponential function. On the other hand, both the Gamma and the *q*-generalized Gamma distributions provide excellent fits to the empirical data, while the *q*-exponential distribution deviates at short and large $T^{\prime }$. The good matching of the *q*-generalized Gamma function with the data indicates that seismicity is correlated at all timescales, while the power-law scaling that emerges at short ($∼{T^{\prime }}^{-0.65}$) and large inter-event times ($∼{T^{\prime }}^{-3.45}$) provides good evidence of the multifractal character of the seismic activity at the West Corinth rift [87].

Investigation of the spatio-temporal properties of the 2003 Lefkada aftershock sequence was reported by Vallianatos et al. [88]. They calculated the inter-event spatial distance *D* as the three-dimensional Euclidean distance between successive earthquake locations $D=\left|\right|r_{k+1}-r_{k}\left|\right|$. They used the cumulative distribution function given by Equation (22) for the inter-event times and distances. The mainshock epicenters and fault plane solutions of the 2003 Lefkada aftershock sequence were separated into four clusters (see their Figure 2a). The analysis resulted in values of $q_{T}=1.395$, 1.47, 1.28, 1.16 and $q_{D}=0.64$, 0.54, 0.77, 0.425 for each cluster, with values of the sum $q_{T}+q_{D}=2.03$, 2.01, 2.05 and 1.58, respectively. These values are also supportive of the conjecture $q_{T}+q_{D}≲2$ proposed by Abe and Suzuki [33]. A study of the distribution of inter-event times of seismic events in Greece, between 1976 and 2009, for different magnitude thresholds using the distribution function

(66) $$P_{M}\left(T\right)=\frac{C}{{\left[1+(q-1)\beta T\right]}^{1/(q-1)}},$$

was carried out by Antonopoulos et al. [68], where *C* is a normalization constant and the parameters β and *q* depend on the fixed mean inter-event time $\tau_{M}$. For the entire dataset (raw catalog) and earthquake magnitudes $M\geq M_{c}$, Equation (66) provided a good fit to the data for $q_{T}=1.24\pm 0.054$, while for the corresponding declustered set good fitting to the observed data was achieved for $q_{T}=1.14\pm 0.057$. Evidently, the inclusion of aftershocks increases the *q*-value in the inter-event distribution (see their Figure 2a). This also suggests that main earthquakes accompanied by their aftershocks are more strongly time-correlated. These authors employed the *hazard function*, $W_{M}$, given by the expression [89]

(67) $$W_{M}\left(T,\Delta T\right)=\frac{\int_{T}^{T+\Delta T}P_{M}\left(t\right)dt}{\int_{T}^{\infty }P_{M}\left(t\right)dt}=1-{\left[1+\frac{\beta (q-1)\Delta T}{1+\beta (q-1)T}\right]}^{\left(q-2)/(q-1\right)},$$

and defined as the probability that at least one earthquake with magnitude $M\geq M_{c}$ occurs in the next time interval ΔT if the last earthquake occurred *T* days ago. Since for distribution functions decaying as a power law $W_{M}\left(T,\Delta T\right)\propto \Delta T/T$ for ΔT≪T [68], it follows that, for small ΔT, the probability of occurrence of at least one earthquake decreases as *T* increases. If, on the other hand, ΔT increases the probability of earthquake occurrence increases.

**Figure 9:**
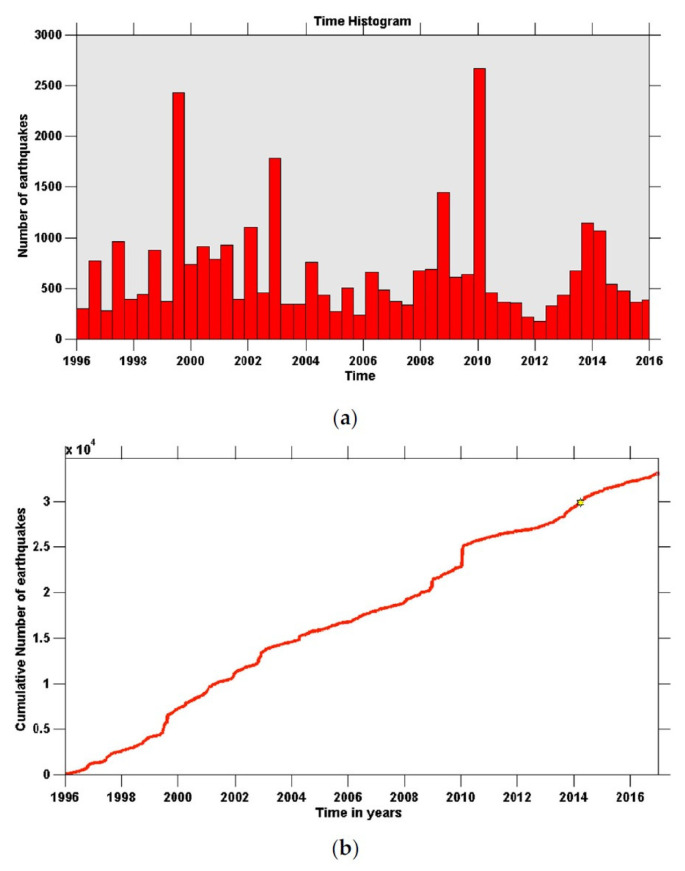
(**a**) Seismicity rate and (**b**) cumulative number of earthquakes in the Yellowstone volcanic field from 1996 to 2016. All events with $M>M_{c}=1.5$ are included. From Chochlaki et al. [41] (their Figure 2). (Online version in color).

More recently, Chochlaki et al. [41] examined the spatio-temporal scaling properties of both the 2008–2009 Yellowstone Lake and the 2010 Madison Plateau earthquake swarms, using the cumulative *q*-exponential distribution of inter-event distances, *P*(>*D*), and times, *P*(>*T*), between successive earthquakes, as given by Equation (22). These authors found that the *q*-exponential distribution described the datasets for both swarms very well. The best fittings for the 2008–2009 Yellowstone Lake swarm corresponded to values of $q_{T}=1.715\pm 0.02$ and $q_{D}=0.710\pm 0.04$, while for the 2010 Madison Plateau earthquake swarm the best fittings were obtained for $q_{T}=1.745\pm 0.065$ and $q_{D}=0.517\pm 0.036$. The seismicity rate and the cumulative number of earthquakes in the Yellowstone volcanic field during the period between 1996 and 2016 for all events with magnitude $M>M_{c}=1.5$ are displayed in Figure 9. Coincident with the arrival of low-frequency, large-amplitude surface waves of the 2002 $M_{w}=7.9$ Denali fault earthquake in Alaska, the Yellowstone National Park region has been undergoing an abrupt increase of seismic events, in spite of the large epicentral distance of 3100 km [90].

### 3.5. Plate Tectonics as a Sub-Extensive System

The question of whether plate tectonics is described by Tsallis statistics was first considered by Vallianatos and Sammonds [91]. According to McKenzie and Parker [92], plate tectonics refers to the slow motion of large lithospheric plates (i.e., large pieces of the Earth’s surface), driven by convection cells deep in the mantle. In 2003, Bird [93] introduced the idea that tectonic plates follow a power-law distribution, which fits well with a fractal and self-organized system [94,95]. Therefore, the application of non-extensive statistics to these systems is appropriate, since non-linearity, long-range interactions, fractality, self-organized criticality, long memory effects and scaling are all of concern. The cumulative frequency distribution of the areas of tectonic plates as a function of the tectonic plate area (in steradians, sr) follows a power law with exponent μ=1/3 (i.e., $F\left(>A\right)\propto A^{1/3}$) between the crossover points $A_{c1}\approx 0.002$ sr and $A_{c2}\approx 1$ sr, as depicted in Figure 1 of Vallianatos and Sammonds [91]. Based on this observation, these authors employed the crossover formulation of the Tsallis *q*-entropy to calculate the probability density function for the areas of the tectonic plates, which could be obtained by solving Equation (57) with r=1 and $z=A_{i}$, where $A_{i}$ denotes all possible values (i.e., microstates) of the tectonic plate areas. In the case where $\beta_{q}\gg \beta_{1}$, Equation (58) splits up into the three asymptotic solutions

(68) $$\begin{array}{ccc} p(A_{i}) & \propto & 1-\beta_{q}A_{i}\;\mathrm{for}\;0\leq A_{i}\leq A_{c1}, \end{array}$$

(69) $$\begin{array}{ccc} p(A_{i}) & \propto & {\left[\left(q-1\right)\beta_{q}\right]}^{1/(1-q)}A_{i}^{1/(1-q)}\;\mathrm{for}\;A_{c1}\leq A_{i}\leq A_{c2}, \end{array}$$

(70) $$\begin{array}{ccc} p(A_{i}) & \propto & {\left(\frac{\beta_{1}}{\beta_{q}}\right)}^{1/(q-1)}\exp\left(-\beta_{1}A_{i}\right)\;\mathrm{for}\;A_{1}\geq A_{c2}, \end{array}$$

where $A_{c1}=1/\left[\left(q-1\right)\beta_{q}\right]$ and $A_{c2}=1/\left[\left(q-1\right)\beta_{1}\right]$.

The power law of *F*(>*A*) between the crossover points $A_{c1}$ and $A_{c2}$ suggests an asymptotic solution of the form given by Equation (69), i.e.,

(71) $$p\left(A_{i}\right)∼A_{i}^{-(1+\mu )}.$$

A comparison with the exponent of $A_{i}$ in Equation (69) yields q=1.75, which is very similar to the *q*-values for earthquakes. This result supports the conclusion that plate tectonics behaves as a sub-extensive (q>1) system. From the expressions of the crossover points given above, Vallianatos and Sammonds [91] obtained that $\beta_{q}\approx 650$ and $\beta_{1}\approx 4/3$ were consistent with the assumption that $\beta_{q}\gg \beta_{1}$. The plate tectonics model based on non-extensive statistics predicts the following separation of scales for the plate areas: (a) a form of p(A) given by Equation (68) is predicted at small scales; (b) a power-law distribution for p(A) of the form given by Equation (69) is predicted at intermediate scales, where the interactions are stronger; (c) a memory-less BG statistics for P(A) is predicted at larger scales, according to Equation (70), which involves only a few major plates.

## 4. Non-Extensivity and Natural Time

A procedure that combines the non-extensive extension of the GR law with natural time and detrended fluctuation analysis (DFA) was first introduced by Sarlis et al. [42] to analyze real seismic data of California and Japan. These authors proved that the combination of non-extensivity with natural time analysis is an improved methodology, that can be applied to examine the observed seismic data fluctuations. In particular, natural time was introduced in 2001 by Varotsos and co-workers [96,97,98] and its strength lies in revealing hidden dynamical features in the time series of complex systems and in identifying when such systems are approaching criticality, which occurs when the variance of natural time, namely $\langle \chi^{2}\rangle -{\langle \chi \rangle }^{2}=\kappa_{1}=0.070$ [96,97,98,99,100,101,102]. A further important feature of natural time is that it allows the origin of self-similarity in signals emitted by complex systems to be distinguished [103]. In a time series consisting of *N* events, the natural time $\chi_{k}=k/N$ can be used as an index for the occurrence of the *k*th event [96,97], as a result of its definition $\chi_{k}\leq 1$. On the other hand, DFA is a powerful technique for the detection of long-range, power-law correlations that are embedded in non-stationary signals [104,105]. For details of how the method works and its application to complex systems the reader is referred to References [106,107]. Here we limit ourselves to saying that at the end of the detrended process the self-similarity exponent, $\alpha_{\mathrm{DFA}}=\alpha$, is obtained, which represents the long-range, power-law correlations of the analyzed signal. In general, if α=0.5 the signal is uncorrelated (white noise). If, on the other hand, α<0.5 the signal is anticorrelated, while if α>0.5 the signal is correlated. The case when α=1.5 corresponds Brownian motion (i.e., integrated white noise).

In the analysis of seismicity the evolution of the natural time $\chi_{k}$ along with the seismic energy released, $E_{k}$, during the *k*th event is important. In terms of the natural frequency, ω=2πϕ, the continuous function Φ(ω) was introduced [96,97]

(72) $$\Phi \left(\omega \right)=\sum_{k=1}^{N}p_{k}\exp\left(i\omega \chi_{k}\right),$$

where

(73) $$p_{k}=\frac{E_{k}}{\sum_{j=1}^{N}E_{j}},$$

so that a normalized power spectrum can be obtained as $\Pi \left(\omega \right)={\left|\Phi \left(\omega \right)\right|}^{2}$, which is a characteristic function for the probability distribution $p_{k}$. Once the behavior of this function is known to be around ω=0, the moments of the distribution and the distribution itself can be determined. As long as ω→0, $\Pi \left(\omega \right)\approx 1-\kappa_{1}\omega^{2}$. Hence, as ω→0, the variance of the natural time, $\kappa_{1}$, has been shown to be an order parameter for seismicity [108,109]. In Sarlis et al.’s [42] analysis, the probability density funcion $P(\kappa_{1})$ was plotted against $\kappa_{1}$, resulting from the natural time analysis of temporally uncorrelated data obtained from Equation (48), for four different values of *q* (=1.62, 1.64, 1.65, 1.68) (see their Figure 2), lying in the universal range 1.6≤q≤1.7 suggested by Vilar et al. [72]. The synthetic data obeying Equation (48) was compared with the observed data for the seismicity of southern California and Japan. An interesting feature emerging from this plot was that the synthetic data differed from the real data, implying that temporal correlations did exist in the real seismic data and, therefore, the GR law could not fully account for the complexity of seismic events [108]. These authors also noted that the existence of temporal correlations emerges when a DFA is applied to the magnitude time series of the real data, revealing a DFA exponent α≈ 0.6 for short scales and α = 0.8–0.9 for longer scales. When these values were inserted into the synthetic data coming from either the GR law or Equation (48), the resulting curves were in much better agreement with the observed seismic data of Japan and southern California, stressing the relevance of long-range temporal correlations between the magitudes of successive earthquakes. In view of these results, it was concluded that the *q*-index by itsef could not be used as a measure of temporal organization, since it did not capture the effects of long-range temporal correlations between the magnitudes of successive events.

Based on the observation that earthquakes are critical phenomena, in a recent work Varotsos et al. [110] investigated whether earthquakes conform with the Lifshitz–Slyozov–Wagner (LSW) theory of dynamic phase transitions by performing a natural time analysis of Japan seismicity. When a mainshock, i.e., the new phase occurs, the order parameter $\kappa_{1}$ changes abruptly. A dynamic entropy, *S*, in natural time is defined according to

**Figure 10:**
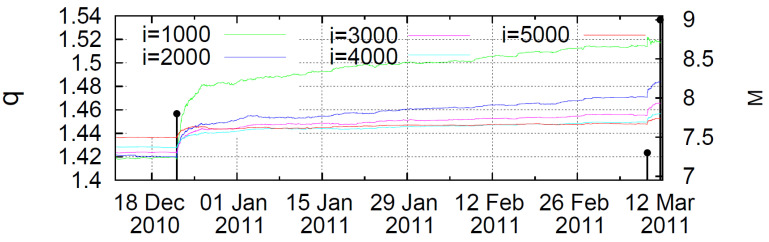
Time variation of the entropic *q*-index at several sliding windows after the occurrence of the M7.8 earthquake on 22 December 2010. A precursory increase of *q* is observed prior to the Tohoku M9.0 mega-earthquake on 12 March 2011. From Varotsos et al. [110] (their Figure 7). (Online version in color).

(74) S=〈χlnχ〉−〈χ〉ln〈χ〉,

where $\langle f\left(\chi \right)\rangle =\sum_{k=1}^{N}p_{k}f\left(\chi_{k}\right)$. Upon reversing the time arrow and applying the time reversal operator to the time series, i.e., $Tp_{k}=p_{N-k+1}$, the value of the entropy changes and it will be denoted by $S_{-}$ [111]. Now, using the complexity measure

(75) $$\Lambda_{i}=\frac{\sigma (\Delta S_{i})}{\sigma (\Delta S_{100})},$$

where *i* is the number of consecutive earthquakes (i.e., the length of a moving window sliding through the time series of consecutive events), $\Delta S_{i}=S_{i}-{\left(S_{-}\right)}_{i}$ is the change of entropy under time reversal and $\sigma (\Delta S_{i})$ is the standard deviation of $\Delta S_{i}$, Varotsos et al. [110] examined the seismicity in Japan by taking into account all earthquakes with M≥3.5 from 1984 until the Tohoku earthquake on 11 March 2011. In relation (75) the denominator was chosen to correspond to the standard deviation of the time series of $\Delta S_{i}$ of 100 events. The results of this analysis revealed that almost two and half months before the M9 earthquake, the $\Lambda_{i}$ values changed abruptly for all the scales observed. After the M7.8 earthquake on December, 2010, the complexity measure showed a scaling behavior with time, $\Delta \Lambda_{i}=C{\left(t-t_{0}\right)}^{a}$, with a≈1/3 independently of *i*. The factor C∝i and $t_{0}\approx 0.2$ days after the M7.8 earthquake occurrence. This result implies that earthquakes conform with the LSW theory on phase transitions, where the time growth of the characteristic size of the minority phase droplets follows $t^{1/3}$. Based on the earthquake magnitude distribution given by Equation (48), Varotsos et al. [110] derived the time variation of the entropic *q*-index during the period 1984–2011. In particular, Figure 10 shows the time variation of *q* at different sliding windows from 10 December 2010, until the Tohoku earthquake on 11 March 2011. From this figure it is clear that the entropic index exhibited a precursory increase prior to the M9 mainshock.

## 5. Non-Extensivity in Precursory Electromagnetic Anomalies

The recording of the pre-seismic kHz to MHz electromagnetic emissions produced by opening cracks, due to mechanical loading at a geophysical scale are precursors of the fractures that occur in the focal areas before the final break-up, which gives rise to an earthquake [112,113,114,115]. In this sense, earthquakes are nothing more than large-scale fractures of the highly heterogeneous system that surrounds the family of asperities. In particular, in pre-seismic fracturing the precursory electromagnetic MHz emissions appear earlier than the kHz radiation. Analyses of pre-seismic MHz-kHz electromagnetic fluctuations that have been conducted by means of critical phenomena indicate that there is a possible two stage transition from the normal state to the catastrophic seismic event [112,113]. Using a Tsallis-like time-dependent entropy formulation, Kalimeri et al. [43] focused on the finally emerged kHz signals to investigate whether new signatures further indicate transition to the last stage of the earthquake preparation process, i.e., the fracture of the main asperities. In order to do so, they concentrated on the candidate kHz signal precursors associated to the Athens and Kozani–Grevena earthquakes, with particular focus on the precursors of the 7 Sepetember 1999, M5.9 Athens earthquake. For reference, the Kozani–Grevena earthquake occurred on 13 May 1995, with magnitude M=6.5.

**Figure 11:**
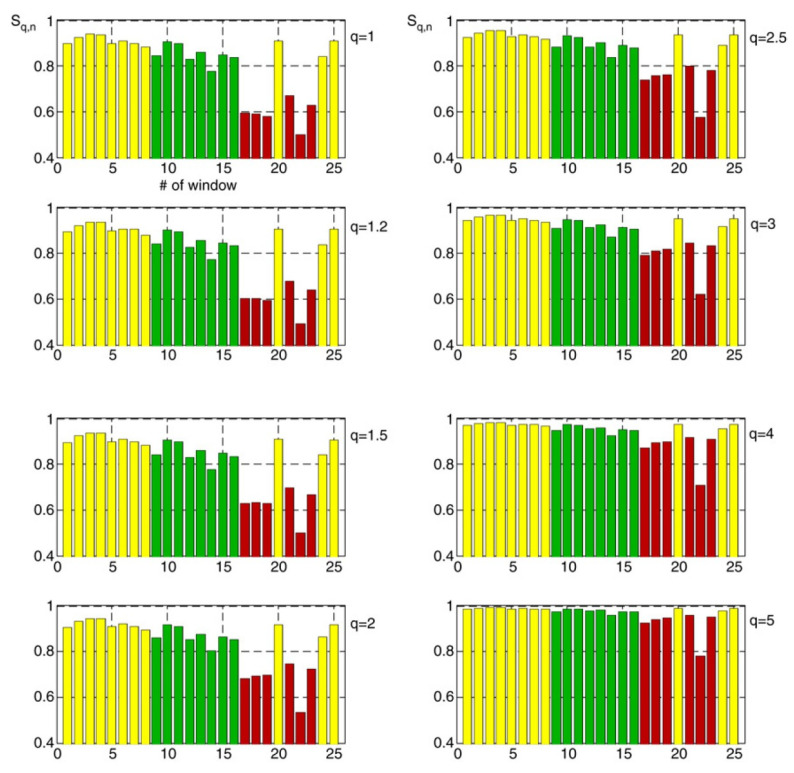
Normalized Tsallis entropy, $S_{q}$, for 25 segments of the time series of the 10 kHz magnetic field strength associated with the 7 Sepetember 1999 M5.9 Athens earthquake for varying values of the *q*-index. The yellow bars indicate the values of entropy far from the time of earthquake occurrence, while the green and red bars with lower entropies are indicative of higher degrees of organization and correspond to values of the entropy closer to the earthquake occurrence. From Kalimeri et al. [43] (their Figure 4). (Online version in color).

The time evolution of the Tsallis entropy was quantified by segmenting the time series of the 10 kHz magnetic field strength associated with the Athens earthquake into 25 time windows, and by expressing the Tsallis entropy in terms of symbolic dynamics (for details on the form of the entropy and segmentation of the time series see Section 4 and Figure 3 of Ref. [43]). Kalimeri et al. [43] calculated the entropy for each of the time windows for q=1.0, 1.2, 1.5, 2.0, 2.5, 3.0, 4.0 and 5.0. Figure 11 shows the behavior of the Tsallis entropy for all 25 windows and each value of *q*. The windows from 1 to 8 (yellow bars) are far from the time of earthquake occurrence and, therefore, the associated entropies characterize the electromagnetic background noise in the region of the station. Such high values of $S_{q}$ are indicative of a low degree of organization. Conversely, the green windows from 9 to 16 are indicative of a higher degree of organization. as their associated entropies point to lower values. The red windows from 17 to 19 and from 21 to 23 have correspondingly lower entropies and, therefore, are indicative of a much higher degree of organization. A look at Figure 11 clearly shows that the values of the entropic index restricted to the interval 1<q<2 provide a superior description of the increasing organization as the earthquake was approaching. This finding clearly demonstrates the sub-extensivity of the underlying fracto-electromagnetic mechanism and is in complete agreement with the upper limit q<2 that was observed in almost all studies of non-extensive seismology.

In a further work Contoyiannis and Eftaxias [116] focused their attention to the earlier pre-seismic MHz signals, by combining ideas of the Tsallis and Lévy statistics, having characteristic function of the form [117]

(76) $$G\left(k\right)=\exp\left(-{c|k|}^{a}\right),$$

where 0<a<2. In particular, the MHz electromagnetic time series can be described in analogy with a thermal second-order phase transition. For a dynamical system described by the intermittent map

(77) $$y_{n+1}=y_{n}+wy_{n}^{m},$$

for w>0 and m>1, the relation between the entropic index *q* and the Lévy index *a* is given by [118]

(78) $$q=\frac{2+a}{1+a}.$$

Contoyiannis and Eftaxias [116] investigated the pre-seismic electromagnetic time series in terms of criticality, based on the method of critical fluctuations [119], described through the statistical distribution of laminar lengths *l* for the map [120]

(79) $$P\left(l\right)∼l^{-p_{l}}.$$

Determination of the exponent $p_{l}$ is at the heart of the method of critical fluctuations. The distribution of the laminar lengths is fitted by the function $R\left(s\right)∼s^{-p_{2}}\exp\left(-p_{3}s\right)$. In conditions of criticality the exponent $p_{3}\to 0$ and, therefore, $p_{2}=p_{l}>1$ [119]. The relation between the Lévy exponent *a* and the critical fluctuation exponent $p_{2}$ is given by $p_{2}=1+a$. Using this relation, together with Equation (78), yields

(80) $$q=1+\frac{1}{p_{2}},$$

which provides an estimation of the Tsallis entropic index for the case of a fracture process in an heterogeneous medium. A relation between the Hurst exponent, *H*, which characterizes the persistent/anti-persistent properties of the signals, and the $p_{2}$ exponent was introduced by Contoyannis et al. [113], where an upper limit of 0.5 for *H* corresponded to the upper limit of 1.5 for $p_{2}$, which, in turn, gave the lower limit of ≈1.66 for the *q*-index, according to Equation (80), suggesting that the *q*-index rooted in a stationary time window, including critical fluctuations, is restricted in the interval 1.66≲q<2. In particular, for the MHz electromagnetic time series associated with the Athens earthquake these authors found a critical exponent $p_{2}=1.3$, which resulted in a *q*-parameter approximately equal to 1.77 well within the restricted interval.

## 6. Conclusions

In this paper we have reviewed some past and recent work dealing with applications of the Tsallis *q*-statistics, which forms the basis of the so-called *non-extensive statistical mechanics*, to the analysis of seismic events. Tsallis entropy is, formally, an extension of the Boltzmann–Gibbs entropy for the description of non-equilibrium stationary states of complex systems whose elements are strongly correlated, which, like the earthquake generation process, typically present a power-law behavior, enhanced by fractal and multifractal geometries, long-range interactions, long-memory effects and self-organized criticality [26,27,28,29,30]. In particular, the index *q* that enters the definition of the Tsallis entropy is a real number that measures the degree of non-extensivity of the system. In reference to the entropy, values of q>1 denote sub-additivity, while q=1 and q<1 correspond to additivity and super-additivity, respectively [26,29].

Although today a full description of seismicity is out of reach, Tsallis theory has provided a suitable framework for the analysis of many aspects of seismicity [10,20,44,63]. It has been central in the description of earthquakes regarding their magnitude–frequency distribution of released earthquake energies [37,38,39,40,41,68,69] as well as in the inter-event time and inter-event distance distributions [35,41,55,87,89], providing the estimation of the Tsallis entropic triplet $\left(q_{M},q_{T},q_{D}\right)$, where $q_{M}$, $q_{T}$ and $q_{D}$ are the entropic indices associated to the magnitude–frequency, inter-event time and inter-event distance distributions, respectively. An important achievement of all these studies is that the *q*-index always lies in the interval 1≤q<2, as obtained for different seismic areas around the world [37,42,43,72,113], thus suggesting a sort of universal character in the non-extensive interpretation of seismicity. On the other hand, $q_{M}$ variations with time have been used as a good indicator of tectonic stability in a seismic area and its proximity to an earthquake [35,37,39,40,43,69,73,75]. For example, in different regions it has been observed that $q_{M}$ variations are strongly related to the energy release prior, and during, an earthquake [69]. Our present understanding of the processes leading to strong catastrophic events has also benefited from the analysis of the spatio-temporal properties of seismicity, including the time evolution of the entropic indices $q_{T}$ and $q_{D}$, where typically $q_{T}>1$ and $q_{D}<1$. As a further universal aspect of seismicity, the results of these analyses suggest the so-called spatio-temporal duality of earthquakes, namely $q_{T}+q_{D}≲2$ [22,74,83]. This result has been verified in laboratory experiments, as well as in numerical models and regional and global seismicity [31,32,33,34,35].

Progress has also been made on the side of combining the non-extensive generalization of the GR law with natural time analysis and detrended fluctuation analysis for the detection of long-range temporal correlations in real seismic data [42,108,110]. Preliminary studies in this line point to the conclusion that the *q*-index cannot be considered a measure of temporal organization unless Tsallis formulation is supplemented by long-range temporal correlations via combination with natural time analysis [42,108]. Moreover, the complexity measure associated with fluctuations of the entropy change of seismicity in natural time under time reversal exhibits an abrupt increase in the proximity of a strong earthquake, conforming with the Lifshitz–Slyozov–Wagner theory for phase transitions [110]. As a final remark, the time evolution of the Tsallis entropy has proved to be a powerful tool for monitoring the focal area states of an impending earthquake [43]. Estimations of the Tsallis index *q* for the fracture processes in heterogeneous media have also indicated that, during precursory electromagnetic activity as correlated with the fracture of strong and large asperities distributed along the activated fault, the *q*-index is restricted to the interval 1.66≲q<2 [113,116]. Further analyses and measurements, as well as the modeling of underlying processes prior to strong events are necessary in order to achieve better short-term earthquake predictions.

## Data Availability

This study did not report any data.
